# Correction to “B‐cell Maturation Antigen‐Based Therapies Post‐Talquetamab in Relapsed or Refractory Multiple Myeloma”

**DOI:** 10.1002/jha2.70056

**Published:** 2025-05-19

Shrestha A, Alzubi M, Alrawabdeh J, Schinke C, Thanendrarajan S, Zangari M, et al. B‐cell maturation antigen‐based therapies post‐talquetamab in relapsed or refractory multiple myeloma. eJHaem. 2024;5:554‐59. doi:10.1002/jha2.896

Carolina Schinke has mistakenly not declared her Conflict of Interest and would like to add a sentence stating “CS has participated in Advisory Board Meetings of Janssen, Pfizer and Arcellx”.

We apologize for this error.

